# Evaluation of Predict, a prognostic risk tool, after diagnosis of a second breast cancer

**DOI:** 10.1093/jncics/pkad081

**Published:** 2023-09-29

**Authors:** Zhengyi Deng, Miranda R Jones, Antonio C Wolff, Kala Visvanathan

**Affiliations:** Stanford School of Medicine, Palo Alto, CA, USA; Johns Hopkins Bloomberg School of Public Health, Baltimore, MD, USA; Department of Oncology, Kimmel Cancer Center, Johns Hopkins School of Medicine, Baltimore, MD, USA; Department of Oncology, Kimmel Cancer Center, Johns Hopkins School of Medicine, Baltimore, MD, USA; Johns Hopkins Bloomberg School of Public Health, Baltimore, MD, USA; Department of Oncology, Kimmel Cancer Center, Johns Hopkins School of Medicine, Baltimore, MD, USA

## Abstract

**Background:**

The UK National Health Service’s Predict is a clinical tool widely used to estimate the prognosis of early-stage breast cancer. The performance of Predict for a second primary breast cancer is unknown.

**Methods:**

Women 18 years of age or older diagnosed with a first or second invasive breast cancer between 2000 and 2013 and followed for at least 5 years were identified from the US Surveillance, Epidemiology, and End Results (SEER) database. Model calibration of Predict was evaluated by comparing predicted and observed 5-year breast cancer–specific mortality separately by estrogen receptor status for first vs second breast cancer. Receiver operating characteristic curves and areas under the curve were used to assess model discrimination. Model performance was also evaluated for various races and ethnicities.

**Results:**

The study population included 6729 women diagnosed with a second breast cancer and 357 204 women with a first breast cancer. Overall, Predict demonstrated good discrimination for first and second breast cancers (areas under the curve ranging from 0.73 to 0.82). Predict statistically significantly underestimated 5-year breast cancer mortality for second estrogen receptor–positive breast cancers (predicted-observed = ‒6.24%, 95% CI = ‒6.96% to ‒5.49%). Among women with a first estrogen receptor–positive cancer, model calibration was good (predicted-observed = ‒0.22%, 95% CI = ‒0.29% to ‒0.15%), except in non-Hispanic Black women (predicted-observed = ‒2.33%, 95% CI = ‒2.65% to ‒2.01%) and women 80 years of age or older (predicted-observed = ‒3.75%, 95% CI = ‒4.12% to ‒3.41%). Predict performed well for second estrogen receptor–negative cancers overall (predicted-observed = ‒1.69%, 95% CI = ‒3.99% to 0.16%) but underestimated mortality among those who had previously received chemotherapy or had a first cancer with more aggressive tumor characteristics. In contrast, Predict overestimated mortality for first estrogen receptor–negative cancers (predicted-observed = 4.54%, 95% CI = 4.27% to 4.86%).

**Conclusion:**

The Predict tool underestimated 5-year mortality after a second estrogen receptor–positive breast cancer and in certain subgroups of women with a second estrogen receptor–negative breast cancer.

Effective screening and treatment strategies have led to a reduction in breast cancer mortality. As a consequence, many patients with breast cancer can survive long enough to develop a second cancer. It has been estimated that approximately 20% of breast cancer survivors will develop a second primary cancer within 25 years of diagnosis, of which 40% will be breast cancers (1,2). Prior studies by our group have shown that breast cancer survivors who developed a second breast cancer experienced statistically significantly higher mortality than women with a first breast cancer, and the mortality is highest among non-Hispanic Black survivors (3,4).

The UK National Health Service’s Predict is an online prognostic tool that can generate individualized prediction of breast cancer–specific and all-cause mortality for women diagnosed with early-stage invasive breast cancer (5,6). It is used to help oncology clinicians provide survival information to patients and make treatment decisions. The Predict online tool has been widely used across the world, with 20 000 visits per month (6). It has been validated for primary breast cancers in multiple female populations from Europe, Canada, and Southeast Asia (7-11). There are no specific prognostic tools to assist clinician-patient decision making after a second breast cancer, however, and the clinical accuracy of existing tools such as Predict is unknown.

In this study, we evaluated the performance of the Predict tool in calculating the 5-year breast cancer–specific mortality among women diagnosed with a second breast cancer using data from the Surveillance, Epidemiology, and End Results (SEER) program and compared the predictive accuracy of this model with that of women diagnosed with a first breast cancer during the same time period.

## Methods

### Study population

The study population was from the SEER 18 registry (2000-2018) (12). SEER is a US program that collects information about patients with cancer from cancer registries across the country. SEER 18 covers 18 cancer registries, representing 28% of the US population. No approval was needed for this study as data were publicly available from SEER program.

SEER collected information on multiple primary cancers and distinguishes them from recurrences based on a series of rigorous criteria, such as the cancer site (*International Classification of Diseases for Oncology, 3rd Edition* topography codes), time since initial cancer diagnosis, tumor histology, tumor behavior, and laterality for paired organs (13).

From the SEER 18 database, we identified 2 cohorts of women 18 years of age or older diagnosed with invasive breast cancer. The first cohort included women diagnosed with 1 incident local or regional breast cancer treated by surgery between January 1, 2000, and December 31, 2013. The second cohort included women diagnosed with both an incident breast cancer of any stage and a subsequent local or regional breast cancer treated by surgery during the same time period. To minimize misclassification between synchronous and subsequent cancers, second breast cancer was defined as a breast cancer diagnosed at least 1 year after the first breast cancer (14). Women in both groups were followed until December 31, 2018, to ensure at least 5 years of follow-up. We excluded women who developed more than 2 cancers during the study period.

### Model covariates

The Predict model incorporates information about age at diagnosis (year), tumor size (mm), tumor grade (1: well differentiated, 2: moderately differentiated, 3: poorly or undifferentiated), number of positive lymph nodes, estrogen receptor status (positive or negative), HER2 status (positive, negative, or unknown), KI-67 status (positive, negative, or unknown), mode of detection (screen or clinical), hormone therapy (yes or no), chemotherapy (no, second generation, third generation), trastuzumab use (yes or no), and bisphosphonate use (yes or no).

In SEER data, HER2 status was available only after 2010, and KI-67 status was not available. Therefore, missing values for HER2 and KI-67 status were entered as “unknown,” as allowed by the Predict model. For mode of detection, we assumed that 70% of breast cancers were detected by screening, based on previous studies among US women (15,16). Of note, mode of detection influenced the mortality prediction of estrogen receptor–positive cancers only, not estrogen receptor–negative cancers.

Details of the chemotherapy drugs given to patients were not available in SEER. Therefore, we classified “second generation” as chemotherapy treatments reported before 2005 and “third generation” as chemotherapy treatments reported during 2005 or later. The SEER chemotherapy variable was categorized as “yes” (patient had chemotherapy) and “no/unknown” (no evidence of chemotherapy was found in the medical records examined) to reflect the fact that the cancer registries were unable to capture complete treatment information based on medical records. Chemotherapy is less likely to have been used on early-stage estrogen receptor–positive diseases because hormone therapy is often the primary treatment (17). Because the majority of women with estrogen receptor–negative disease are treated with chemotherapy, we restricted our analysis to the 70% who received chemotherapy. The following treatment assumptions were made in our primary analysis: 1) all women with estrogen receptor–positive disease received hormone therapy, 2) 70% of women with HER2-positive disease received trastuzumab (18), and 3) no patient received bisphosphonate.

Variables in SEER that were not inputs of the Predict model included race and ethnicity (non-Hispanic White, non-Hispanic Black, non-Hispanic American Indian or Alaska Native, non-Hispanic Asian or Pacific Islander, and Hispanic), tumor histology, and radiation therapy (yes, no/unknown).

### Outcome ascertainment

Death from breast cancer was identified based on the SEER variable “COD to site recode.” Vital status, survival time, and cause of death were ascertained from the National Center for Health Statistics. Women with missing survival time or cause of death (1% of the study population) were excluded.

### Statistical analysis

Demographic and clinical characteristics of first and second breast cancers were summarized by reporting the proportions for categorical variables and mean (SD) for continuous variables. Predict, version 2.1 (19) was used to generate predicted 5-year breast cancer–specific mortality risk for each patient. For the first cancer cohort, mortality estimates were calculated from the first cancer diagnosis. For the second cancer cohort, mortality estimates were generated from the second cancer diagnosis.

The performance of Predict was assessed by examining both model discrimination and calibration. All analyses were conducted separately for the first and second breast cancer cohorts and stratified by estrogen receptor status, given differences in their outcome. The model discrimination was assessed by plotting the receiver operating characteristic curve and calculating the area under the curve (AUC), with further stratification by race and ethnicity. Model calibration was evaluated by comparing the predicted 5-year breast cancer mortality to the observed mortality over the same period. The number of predicted deaths from breast cancer was calculated by summing the predicted scores of each patient generated from the Predict model. Breast cancer mortality was calculated as follows:


$$\frac{Number\;of\;breast\;cancer\;deaths}{Number\;of\;patients\;with\;breast\;cancer}\times 100.$$

We plotted the observed mortality with 95% confidence intervals (CIs) against deciles of the predicted mortality. These plotted estimates were visually compared with the perfect agreement line (y = x). We also quantified the difference between predicted and observed mortality. The relative ratio (predicted mortality divided by the observed mortality) as well as absolute difference (predicted mortality ‒ observed mortality) were calculated. Bootstrap methods were used to generate 95% confidence intervals for relative and absolute differences. A similar analysis was conducted for multiple subgroups based on demographic and clinical characteristics. Further, among women with a second cancer, the difference between predicted and observed mortality for the second cancer was evaluated in subgroups defined by characteristics of their first breast cancer, including age at diagnosis of first cancer, tumor grade, tumor stage, lymph node status, estrogen receptor status, treatments (chemotherapy and radiation therapy), and time between first and second cancer (<5, 5-10, ≥10 years). For the second cancer cohort, we also generated the Predict score from their first cancer diagnosis (only for local or regional-stage cancer treated by surgery) and conducted the analysis stratified by quartiles of this score.

Sensitivity analyses were conducted to check the robustness of some assumptions made by evaluating model performance under alternative situations. The type of chemotherapy was set to second or third generation for all calendar years instead of second generation before 2005 and third generation in 2005 and later. In another analysis, we assumed that less breast cancers (ie, 50%) were detected by screening.

All analyses were performed in R, version 4.1.2 (R Foundation for Statistical Computing, Vienna, Austria).

## Results

### Demographic and clinical characteristics

The study population included 6729 women diagnosed with a second breast cancer (5606 women with estrogen receptor–positive second cancer and 1123 women with estrogen receptor–negative second cancer) as well as 357 204 women with a first breast cancer (303 837 women with estrogen receptor–positive cancer and 53 367 women with estrogen receptor–negative cancer). Table 1 summarizes the baseline characteristics of the study population, stratified by first or second breast cancer and estrogen receptor status. The majority of breast cancers were local and node negative, with a tumor size under 20 mm. As expected, women with a second breast cancer were older than women with a first breast cancer for both estrogen receptor–positive cancers (65 vs 59.7 years of age) and estrogen receptor–negative cancers (56.8 vs 53.2 years of age). Among women with 2 breast cancers, more than 40% of the second tumors were developed within 5 years of the first cancer. Women with a second cancer were more likely to have estrogen receptor–positive than estrogen receptor–negative disease. Close to 23% of women with a second estrogen receptor–negative cancer were non-Hispanic Black compared with 9.4% among women with a second estrogen receptor–positive cancer. For women with a second estrogen receptor–positive cancer, 72% of their first cancers were also estrogen receptor positive; for women with a second estrogen receptor–negative cancer, 45.7% of their first cancers were estrogen receptor negative.

**Table 1.** Baseline characteristics of first and second breast cancers, by estrogen receptor status

	First breast cancer		Second breast cancer	
	Estrogen receptor positive	Estrogen receptor negative	Estrogen receptor positive	Estrogen receptor negative
	(n = 303 837)	(n = 53 367)	(n = 5606)	(n = 1123)
Age of diagnosis, mean (SD), y	59.7 (13.1)	53.2 (11.8)	65.0 (12.4)	56.8 (11.4)
Follow-up time, mean (SD), y	9.66 (4.21)	9.19 (4.83)	7.45 (3.39)	7.30 (3.72)
Year of diagnosis, No. (%)				
2000-2004	86 869 (28.6)	16 896 (31.7)	535 (9.5)	102 (9.1)
2005-2009	111 613 (36.7)	20 562 (38.5)	2049 (36.6)	491 (43.7)
2010-2013	105 355 (34.7)	15 909 (29.8)	3022 (53.9)	530 (47.2)
Race and ethnicity, No. (%)				
Hispanic	28 329 (9.3)	6204 (11.6)	433 (7.7)	135 (12.0)
Non-Hispanic Black	24 403 (8.0)	9207 (17.3)	527 (9.4)	253 (22.5)
Non-Hispanic White	224 417 (73.9)	33 357 (62.5)	4225 (75.4)	652 (58.1)
Other^a^	25 753 (8.5)	4479 (8.4)	419 (7.5)	82 (7.3)
Missing	935 (0.3)	120 (0.2)	2 (0.0)	1 (0.1)
Tumor stage, No. (%)				
Local	199 362 (65.6)	28 799 (54.0)	4142 (73.9)	720 (64.1)
Regional	104 475 (34.4)	24 568 (46.0)	1464 (26.1)	403 (35.9)
Tumor grade, No. (%)				
1	80 328 (26.4)	706 (1.3)	1575 (28.1)	20 (1.8)
2	147 608 (48.6)	8233 (15.4)	2707 (48.3)	207 (18.4)
3	75 901 (25.0)	44 428 (83.2)	1324 (23.6)	896 (79.8)
Lymph node status, No. (%)				
Negative	204 106 (67.2)	30 813 (57.7)	4254 (75.9)	753 (67.1)
Positive	99 731 (32.8)	22 554 (42.3)	1352 (24.1)	370 (32.9)
Tumor size, No. (%)				
<10 mm	63 334 (20.8)	3738 (7.0)	1771 (31.6)	146 (13.0)
10-19 mm	121 908 (40.1)	15 602 (29.2)	2244 (40.0)	397 (35.4)
20-49 mm	99 861 (32.9)	27 203 (51.0)	1332 (23.8)	485 (43.2)
≥50 mm	18 734 (6.2)	6824 (12.8)	259 (4.6)	95 (8.5)
Histology, No. (%)				
Ductal carcinoma	223 668 (73.6)	46 655 (87.4)	3892 (69.4)	961 (85.6)
Lobular carcinoma	26 072 (8.6)	447 (0.8)	642 (11.5)	25 (2.2)
Ductal and locular carcinoma	25 605 (8.4)	1006 (1.9)	496 (8.8)	22 (2.0)
Others	28 492 (9.4)	5259 (9.9)	576 (10.3)	115 (10.2)
Chemotherapy, No. (%)				
No/unknown^b^	185 260 (61.0)	0 (0)	4160 (74.2)	0 (0)
Yes	118 577 (39.0)	53 367 (100)	1446 (25.8)	1123 (100)
Radiation therapy, No. (%)				
No/unknown^b^	130 914 (43.1)	22 086 (41.4)	3650 (65.1)	770 (68.6)
Yes	172 923 (56.9)	31 281 (58.6)	1956 (34.9)	353 (31.4)
Predict 5-y breast cancer mortality, mean (SD)	0.04 (0.07)	0.22 (0.16)	0.04 (0.06)	0.19 (0.15)
**Characteristics of the initial breast cancer for patients with a second breast cancer**				
Time between first and second breast cancer, No. (%),y				
1-4	—	—	2552 (45.5)	531 (47.3)
5-9	—	—	2327 (41.5)	491 (43.7)
≥10	—	—	727 (13.0)	101 (9.0)
Age at diagnosis, mean (SD), y	—	—	59.7 (12.5)	51.7 (11.4)
Tumor stage, No. (%)				
Local	—	—	4061 (72.4)	746 (66.4)
Regional	—	—	1423 (25.4)	356 (31.7)
Distant	—	—	53 (0.9)	7 (0.6)
Missing	—	—	69 (1.2)	14 (1.2)
Tumor grade, No. (%)				
1	—	—	1373 (24.5)	104 (9.3)
2	—	—	2274 (40.6)	311 (27.7)
3	—	—	1485 (26.5)	637 (56.7)
Missing	—	—	474 (8.5)	71 (6.3)
Lymph node status, No. (%)				
Negative	—	—	3784 (67.5)	733 (65.3)
Positive	—	—	1320 (23.5)	324 (28.9)
Missing	—	—	502 (9.0)	66 (5.9)
Tumor size, No. (%)				
<10 mm	—	**—**	1292 (23.0)	171 (15.2)
10-19 mm	—	**—**	2026 (36.1)	366 (32.6)
20-49 mm	—	**—**	1515 (27.0)	425 (37.8)
≥50 mm	—	**—**	306 (5.5)	81 (7.2)
Missing	—	**—**	467 (8.3)	80 (7.1)
Estrogen receptor status, No. (%)				
Positive	—	**—**	4035 (72.0)	493 (43.9)
Negative	—	**—**	901 (16.1)	513 (45.7)
Missing	—	**—**	670 (12.0)	117 (10.4)
Predict 5-y breast cancer mortality, mean (SD)^c^	—	**—**	0.07 (0.10)	0.12 (0.13)
Surgery, No. (%)				
No	—	—	140 (2.5)	24 (2.1)
Yes	—	—	5466 (97.5)	1099 (97.9)
Chemotherapy, No. (%)				
No/unknown^b^	—	—	3662 (65.3)	420 (37.4)
Yes	—	—	1944 (34.7)	703 (62.6)
Radiation therapy, No. (%)				
No/unknown^b^	—	—	2496 (44.5)	416 (37.0)
Yes	—	—	3110 (55.5)	707 (63.0)

a “Other” included non-Hispanic Asian and Pacific Islander, non-Hispanic American Indian, and Alaska Native.

b No evidence of chemotherapy or radiation therapy was found in the medical records. It could be that patients did not have the treatment or patients had the treatment, but it was not recorded in medical records.

c This was calculated at time of initial presentation of local or regional stage breast cancer treated by surgery. Data was also available on tumor grade, lymph node status, tumor size, and estrogen receptor status.

### Estrogen receptor–positive diseases

**Figure 1.**
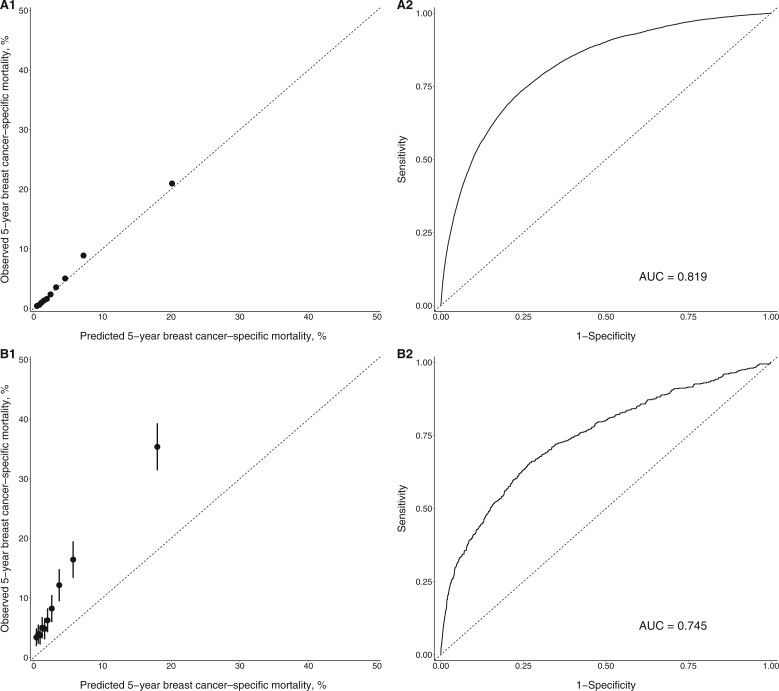
Calibration plot (**A1** and **B1**) and receiver operating characteristic curve (**A2** and **B2**) for estrogen receptor–positive breast cancer. **A1** and **A2** are for first breast cancers. **B1** and **B2** are for second breast cancers. The **dotted line** in **A1** and **B1** indicates perfect agreement between predicted and observed mortality. The **black dots** and **bars** in **A1** and **B1** represent the observed mortality with 95% confidence interval against deciles of the predicted mortality. AUC = area under the curve.

In women with first estrogen receptor–positive breast cancers, the observed mortality agreed with the predicted mortality across all deciles, as shown in the calibration plot displayed in Figure 1, A1. The overall absolute mortality difference was ‒0.22% (95% CI = ‒0.29% to ‒0.15%), and the relative ratio was 0.95 (95% CI = 0.94 to 0.97) (Table 2). The model calibration remained good in most subgroup analyses, as shown in Table 2. The model did, however, underestimate breast cancer mortality in non-Hispanic Black women (absolute difference = ‒2.33%, 95% CI = ‒2.65% to ‒2.01%) and women 80 years of age or older (absolute difference = ‒3.75%, 95% CI = ‒4.12% to ‒3.41%). The overall model discrimination was excellent, with an AUC of 0.819 (Figure 1, A2). The AUC by race and ethnicity ranged from 0.806 to 0.831 (Supplementary Table 1, available online).

**Figure 2.**
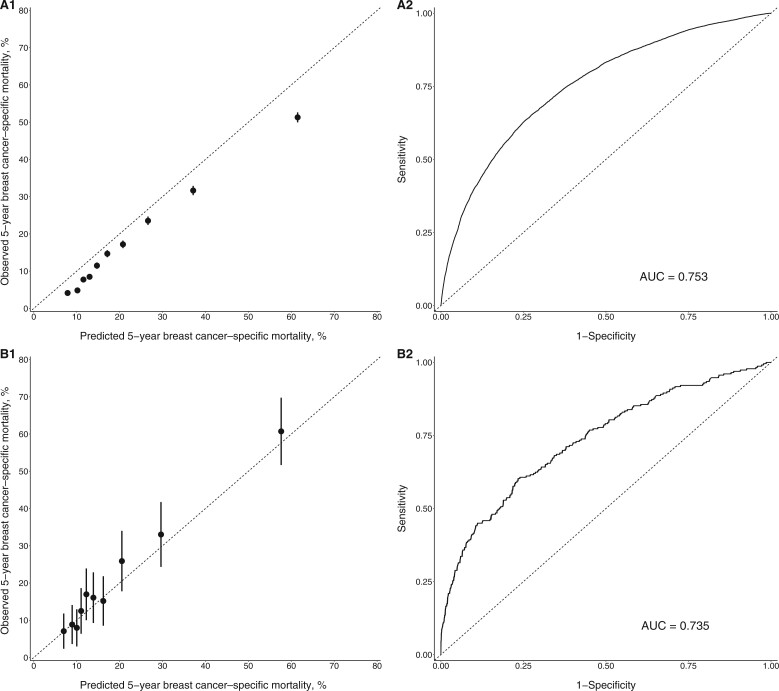
Calibration plot (**A1** and **B1**) and receiver operating characteristic curve (**A2** and **B2**) for estrogen receptor–negative breast cancer. **A1** and **A2** are for first breast cancers. **B1** and **B2** are for second breast cancers. The **dotted line** in **A1** and **B1** indicates perfect agreement between predicted and observed mortality. The **black dots** and **bars** in **A1** and **B1** represent the observed mortality with 95% confidence interval against deciles of the predicted mortality. AUC = area under the curve.

**Table 2.** Difference between predicted and observed mortality among women with estrogen receptor–positive breast cancer

	First breast cancer				Second breast cancer			
	Predicted No. of deaths	Observed No. of deaths	Absolute mortality difference (95% confidence interval)^a^	Relative mortality ratio (95% confidence interval)^b^	Predicted No. of deaths	Observed No. of deaths	Absolute mortality difference (95% confidence interval)^a^	Relative mortality ratio (95% confidence interval)^b^
Overall (%)	13 181	13 855	‒0.22 (‒0.29 to ‒0.15)	0.95 (0.94 to 0.97)	206	556	‒6.24 (‒6.96 to ‒5.49)	0.37 (0.34 to 0.4)
Year								
2000-2004	4368	4742	‒0.43 (‒0.57 to ‒0.3)	0.92 (0.9 to 0.94)	25	72	‒8.79 (‒11.57 to ‒6.18)	0.35 (0.29 to 0.43)
2005-2009	4787	4866	‒0.07 (‒0.18 to 0.04)	0.98 (0.96 to 1.01)	78	230	‒7.42 (‒8.69 to ‒6.12)	0.34 (0.3 to 0.39)
2010-2013	4026	4247	‒0.21 (‒0.32 to ‒0.1)	0.95 (0.92 to 0.97)	103	254	‒5 (‒5.99 to ‒4.07)	0.41 (0.36 to 0.46)
Race and ethnicity								
Hispanic	1475	1408	0.24 (‒0.02 to 0.47)	1.05 (1.0 to 1.1)	19	59	‒9.24 (‒12.36 to ‒6.12)	0.32 (0.26 to 0.43)
Non-Hispanic Black	1372	1941	‒2.33 (‒2.65 to ‒2.01)	0.71 (0.68 to 0.74)	28	77	‒9.3 (‒12.25 to ‒6.65)	0.36 (0.3 to 0.44)
Non-Hispanic White	9191	9582	‒0.17 (‒0.25 to ‒0.09)	0.96 (0.94 to 0.98)	146	384	‒5.63 (‒6.51 to ‒4.78)	0.38 (0.35 to 0.42)
Other^c^	1111	916	0.76 (0.54 to 0.98)	1.21 (1.14 to 1.3)	13	36	‒5.49 (‒8.2 to ‒2.97)	0.36 (0.27 to 0.52)
Age at diagnosis, y								
<50	3824	3251	0.76 (0.62 to 0.91)	1.18 (1.14 to 1.22)	26	77	‒7.35 (‒9.51 to ‒5.24)	0.34 (0.28 to 0.42)
50-59	3124	2972	0.19 (0.06 to 0.33)	1.05 (1.01 to 1.09)	45	134	‒7.57 (‒9.38 to ‒5.94)	0.34 (0.28 to 0.39)
60-69	2792	2813	‒0.03 (‒0.15 to 0.09)	0.99 (0.96 to 1.03)	55	130	‒4.64 (‒6.00 to ‒3.35)	0.42 (0.36 to 0.51)
70-79	2142	2657	‒1 (‒1.18 to ‒0.8)	0.81 (0.78 to 0.84)	47	107	‒4.33 (‒5.7 to ‒3.01)	0.44 (0.37 to 0.53)
≥80	1299	2162	‒3.75 (‒4.12 to ‒3.41)	0.6 (0.58 to 0.62)	34	108	‒10.11 (‒12.59 to ‒7.83)	0.31 (0.26 to 0.38)
Tumor size, mm								
<10	625	567	0.09 (0.02 to 0.16)	1.1 (1.02 to 1.19)	17	88	‒4.01 (‒5.03 to ‒3.00)	0.19 (0.16 to 0.25)
10-19	2906	2553	0.29 (0.21 to 0.37)	1.14 (1.1 to 1.18)	55	154	‒4.41 (‒5.47 to ‒3.48)	0.36 (0.31 to 0.41)
20-49	6455	7579	‒1.13 (‒1.28 to ‒0.96)	0.85 (0.83 to 0.87)	85	223	‒10.36 (‒12.44 to ‒8.52)	0.38 (0.34 to 0.43)
≥50	3195	3156	0.21 (‒0.34 to 0.72)	1.01 (0.98 to 1.04)	50	91	‒15.83 (‒21.18 to ‒10.69)	0.55 (0.47 to 0.64)
Tumor stage								
Local	3980	3794	0.09 (0.03 to 0.15)	1.05 (1.02 to 1.08)	76	242	‒4.01 (‒4.74 to ‒3.35)	0.31 (0.28 to 0.36)
Regional	9201	10 061	‒0.82 (‒1.02 to ‒0.65)	0.91 (0.9 to 0.93)	130	314	‒12.57 (‒14.63 to ‒10.51)	0.41 (0.38 to 0.46)
Tumor grade								
1	923	1003	‒0.1 (‒0.18 to ‒0.03)	0.92 (0.87 to 0.98)	15	78	‒4 (‒5.04 to ‒2.97)	0.19 (0.15 to 0.24)
2	5123	5366	‒0.16 (‒0.25 to ‒0.07)	0.95 (0.93 to 0.98)	84	240	‒5.76 (‒6.78 to ‒4.71)	0.35 (0.31 to 0.4)
3	7136	7486	‒0.46 (‒0.67 to ‒0.28)	0.95 (0.93 to 0.97)	108	238	‒9.82 (‒11.84 to ‒7.91)	0.45 (0.41 to 0.51)
Lymph node status								
Negative	4172	4188	‒0.01 (‒0.07 to 0.05)	1 (0.97 to 1.03)	81	256	‒4.11 (‒4.81 to ‒3.41)	0.32 (0.28 to 0.36)
Positive	9010	9667	‒0.66 (‒0.83 to ‒0.47)	0.93 (0.92 to 0.95)	126	300	‒12.87 (‒14.96 to ‒10.7)	0.42 (0.38 to 0.47)
Histology								
Ductal carcinoma	9662	10 432	‒0.34 (‒0.43 to ‒0.27)	0.93 (0.91 to 0.94)	140	358	‒5.6 (‒6.49 to ‒4.82)	0.39 (0.36 to 0.43)
Lobular carcinoma	1305	1301	0.02 (‒0.25 to 0.26)	1 (0.95 to 1.05)	30	86	‒8.72 (‒11.56 to ‒6.2)	0.35 (0.29 to 0.43)
Ductal and lobular carcinoma	1239	1208	0.12 (‒0.11 to 0.38)	1.03 (0.98 to 1.08)	21	57	‒7.26 (‒10.1 to ‒4.66)	0.37 (0.29 to 0.47)
Chemotherapy	7358	7725	‒0.31 (‒0.45 to ‒0.17)	0.95 (0.93 to 0.97)	77	208	‒9.06 (‒10.79 to ‒7.4)	0.37 (0.33 to 0.43)
Radiation therapy	7209	6609	0.35 (0.26 to 0.42)	1.09 (1.07 to 1.11)	75	174	‒5.06 (‒6.29 to ‒3.93)	0.43 (0.38 to 0.49)

a Absolute mortality difference (%) = predicted mortality ‒ observed mortality.

b Relative mortality ratio = predicted mortality/observed mortality.

c “Other” included non-Hispanic Asian and Pacific Islander, non-Hispanic American Indian, and Alaska Native.

The model performance was not as good for women diagnosed with a second estrogen receptor–positive cancer. Predict underestimated mortality across all deciles, with the greatest difference in performance among the higher deciles (Figure 1, B1). The tool underestimated breast cancer–specific mortality by 6.24% (95% CI = ‒6.96% to ‒5.49%) overall. The relative ratio between predicted and observed mortality was 0.37 (95% CI = 0.34 to 0.4). Poor calibration was observed in all subgroups, with an absolute mortality difference ranging from ‒15.83% to −4% and a relative ratio ranging from 0.19 to 0.55 (Table 2). The underestimation was more pronounced among non-Hispanic Black (absolute difference = ‒9.3%, 95% CI = ‒12.25% to ‒6.65%) and Hispanic women (absolute difference = ‒9.24%, 95% CI = ‒12.36% to ‒6.12%). The model also underestimated breast cancer mortality among women who had a first cancer with more aggressive tumor characteristics, including tumor stage, size, grade, lymph node status, and Predict score. In addition, a shorter time interval between first and second cancers was associated with greater underestimation of mortality (Table 4). The AUC was lower at 0.745 in women with a second estrogen receptor–positive cancer overall (Figure 1, B2). Worse model discrimination was observed among non-Hispanic White women (AUC = 0.727) as well as non-Hispanic Asian and Pacific Islander and non-Hispanic American Indian and Alaska Native women (AUC = 0.697) (Supplementary Table 1, available online).

### Estrogen receptor–negative diseases

An overestimation of cancer-specific mortality was found for women with first estrogen receptor–negative breast cancers and received chemotherapy (absolute difference = 4.54%, 95% CI = 4.27% to 4.86%; relative ratio = 1.26, 95% CI = 1.24 to 1.28) (Table 2). The Predict tool overestimated mortality across all deciles (Figure 2, A1). In subgroup analyses, there was an overestimation of mortality in all subgroups except for women 80 years of age or older, where an underestimation was observed (absolute difference = ‒3.91%, 95% CI = ‒7.05% to ‒0.79%) and women with a grade 1 tumor, where predicted and observed mortalities were similar (absolute difference = ‒0.99%, 95% CI = ‒2.83% to 0.81%) (Table 3). When stratified by race, the absolute difference among non-Hispanic Black women was much smaller than among all other races. The model discrimination was good overall (AUC = 0.753) and by race and ethnicity (AUC ranged from 0.744 to 0.754) (Figure 2, A2 and Supplementary Table 1, available online).

**Table 3.** Difference between predicted and observed mortality among women with estrogen receptor–negative breast cancer who received chemotherapy

	First breast cancer				Second breast cancer			
	Predicted No. of deaths	Observed No. of deaths	Absolute mortality difference (95% confidence interval)^a^	Relative mortality ratio (95% confidence interval)^b^	Predicted No. of deaths	Observed No. of deaths	Absolute mortality difference (95% confidence interval)^a^	Relative mortality ratio (95% confidence interval)^b^
Overall (%)	11 768	9343	4.54 (4.27 to 4.86)	1.26 (1.24 to 1.28)	210	229	‒1.69 (‒3.99 to 0.16)	0.92 (0.83 to 1.01)
Year								
2000-2004	4354	3618	4.36 (3.78 to 4.96)	1.2 (1.17 to 1.24)	23	31	‒7.84 (‒15.97 to 0.2)	0.74 (0.58 to 1.01)
2005-2009	4376	3402	4.74 (4.32 to 5.2)	1.29 (1.25 to 1.32)	94	113	‒3.87 (‒7.5 to ‒0.67)	0.83 (0.72 to 0.97)
2010-2013	3038	2323	4.49 (3.98 to 4.99)	1.31 (1.26 to 1.35)	93	85	1.51 (‒1.83 to 3.87)	1.09 (0.91 to 1.29)
Race and ethnicity								
Hispanic	1422	1164	4.16 (3.27 to 5.09)	1.22 (1.17 to 1.29)	27	34	‒5.19 (‒11.68 to 0.54)	0.79 (0.64 to 1.03)
Non-Hispanic Black	2151	2013	1.5 (0.78 to 2.24)	1.07 (1.03 to 1.11)	53	62	‒3.56 (‒8.72 to 1.12)	0.85 (0.7 to 1.06)
Non-Hispanic White	7187	5557	4.89 (4.52 to 5.26)	1.29 (1.27 to 1.32)	118	118	0 (‒2.85 to 2.56)	1 (0.87 to 1.17)
Other^c^	983	604	8.46 (7.44 to 9.36)	1.63 (1.51 to 1.74)	12	15	‒3.66 (‒11.63 to 4.13)	0.8 (0.57 to 1.42)
Age at diagnosis, y								
<50	4443	3676	3.68 (3.2 to 4.17)	1.21 (1.18 to 1.24)	51	72	‒7.09 (‒11.41 to ‒2.56)	0.71 (0.6 to 0.87)
50-59	3729	2779	5.61 (5.01 to 6.1)	1.34 (1.3 to 1.38)	71	69	0.52 (‒2.7 to 3.87)	1.03 (0.87 to 1.26)
60-69	2317	1730	5.54 (4.85 to 6.21)	1.34 (1.29 to 1.4)	48	48	0 (‒3.65 to 4.19)	1 (0.83 to 1.31)
70-79	1050	900	3.53 (2.35 to 4.63)	1.17 (1.11 to 1.23)	32	34	‒1.36 (‒8.35 to 4.59)	0.94 (0.72 to 1.26)
≥80	229	258	‒3.91 (‒7.05 to ‒0.79)	0.89 (0.81 to 0.97)	7	6	4.55 (‒9.6 to 19.62)	1.17 (0.78 to 3.01)
Tumor size, mm								
<10	390	200	5.08 (4.39 to 5.75)	1.95 (1.73 to 2.25)	15	15	0 (‒4.67 to 4.34)	1 (0.68 to 1.77)
10-19	2222	1461	4.88 (4.44 to 5.28)	1.52 (1.45 to 1.59)	50	50	0 (‒3.33 to 2.83)	1 (0.79 to 1.29)
20-49	6176	5167	3.71 (3.26 to 4.12)	1.2 (1.17 to 1.22)	101	116	‒3.09 (‒6.66 to 0.71)	0.87 (0.76 to 1.04)
≥50	2981	2515	6.83 (5.72 to 7.87)	1.19 (1.15 to 1.22)	44	48	‒4.21 (‒12.02 to 4.9)	0.92 (0.8 to 1.12)
Tumor stage								
Local	3669	2415	4.35 (4.05 to 4.65)	1.52 (1.46 to 1.57)	83	82	0.14 (‒2.31 to 2.35)	1.01 (0.83 to 1.26)
Regional	8099	6928	4.77 (4.24 to 5.33)	1.17 (1.15 to 1.19)	127	147	‒4.96 (‒9.26 to ‒0.78)	0.86 (0.77 to 0.98)
Tumor grade								
1	50	57	‒0.99 (‒2.83 to 0.81)	0.88 (0.71 to 1.13)	1	6	‒25 (‒42.32 to ‒5.37)	0.17 (0.15 to 0.46)
2	1732	1151	7.06 (6.36 to 7.78)	1.5 (1.44 to 1.59)	37	35	0.97 (‒4.25 to 5.34)	1.06 (0.8 to 1.44)
3	9986	8135	4.17 (3.83 to 4.48)	1.23 (1.21 to 1.25)	172	188	‒1.79 (‒4.22 to 0.54)	0.91 (0.82 to 1.03)
Lymph node status								
Negative	4049	2784	4.11 (3.79 to 4.42)	1.45 (1.41 to 1.51)	88	90	‒0.27 (‒2.5 to 1.93)	0.98 (0.82 to 1.2)
Positive	7719	6559	5.14 (4.57 to 5.75)	1.18 (1.15 to 1.2)	122	139	‒4.59 (‒9.22 to ‒0.04)	0.88 (0.78 to 1.00)
Histology								
Ductal carcinoma	10 166	8114	4.4 (4.09 to 4.7)	1.25 (1.23 to 1.28)	175	203	‒2.91 (‒5.4 to ‒0.59)	0.86 (0.77 to 0.97)
Lobular carcinoma	141	112	6.49 (2.87 to 10.49)	1.26 (1.1 to 1.5)	6	5	4 (‒13.28 to 16.66)	1.2 (0.63 to 3.39)
Ductal and lobular carcinoma	287	265	2.19 (-0.49 to 4.66)	1.08 (0.98 to 1.2)	5	6	‒4.55 (‒19.38 to 12.38)	0.83 (0.54 to 2.10)
Radiation therapy	7185	5505	5.37 (4.98 to 5.74)	1.31 (1.28 to 1.33)	79	88	‒2.55 (‒6.91 to 1.71)	0.9 (0.76 to 1.08)

a Absolute mortality difference=predicted ‒ observed.

b Relative mortality ratio=predicted/observed.

c “Other” included non-Hispanic Asian and Pacific Islander, non-Hispanic American Indian, and Alaska Native.

For women diagnosed with a second estrogen receptor–negative cancer, the predicted and observed mortality were similar across deciles, as shown in Figure 2, B1. The absolute difference between the overall predicted and observed mortality was ‒1.69% (95% CI = ‒3.99% to 0.16%), and the relative ratio was 0.92 (95% CI = 0.83 to 1.01) (Table 3). Mortality was underestimated in several subgroups, however, including women diagnosed between 2005 and 2009, women younger than 50 years of age, women with a tumor of regional stage, women with a grade 1 tumor, women with positive lymph nodes, and women with ductal carcinoma (Table 3). Similar to estrogen receptor–positive cancer, the model underestimated breast cancer mortality among women who had a first cancer with more aggressive tumor characteristics, including tumor stage, size, grade, lymph node status, and Predict score (Table 4). A shorter time interval between first and second cancers was also associated with greater underestimation of mortality (Table 4). Overall model discrimination was comparable to that for women with first estrogen receptor–negative cancer (AUC = 0.735), with similar AUCs across race and ethnicity (Figure 2, B2 and Supplementary Table 1, available online).

**Table 4:** . Difference between predicted and observed mortality among women with second breast cancer, by characteristics of their first breast cancer

First breast cancer characteristics	Estrogen receptor–positive second breast cancer				Estrogen receptor–negative second breast cancer			
	Predicted No. of deaths	Observed No. of deaths	Absolute mortality difference (95% confidence interval)^a^	Relative mortality ratio (95% confidence interval)^b^	Predicted No. of deaths	Observed No. of deaths	Absolute mortality difference (95% confidence interval)^a^	Relative mortality ratio (95% confidence interval) ^b^
Age at diagnosis, y								
<50	47	137	‒7.04 (‒8.71 to ‒5.41)	0.34 (0.29 to 0.4)	85	101	‒3.27 (‒6.48 to ‒0.06)	0.84 (0.73 to 1.00)
50-59	56	134	‒5.27 (‒6.61 to ‒3.98)	0.42 (0.36 to 0.49)	62	61	0.29 (‒3.3 to 3.87)	1.02 (0.84 to 1.28)
60-69	50	122	‒4.77 (‒6.19 to ‒3.54)	0.41 (0.35 to 0.48)	44	47	‒1.4 (‒6.18 to 3.85)	0.94 (0.77 to 1.23)
70-79	40	113	‒7.1 (‒9.02 to ‒5.29)	0.35 (0.29 to 0.42)	17	19	‒2.99 (‒13.94 to 6.93)	0.89 (0.63 to 1.38)
≥80	15	50	‒11.33 (‒15.41 to ‒7.79)	0.3 (0.22 to 0.38)	2	1	16.67 (‒18.13 to 26.7)	2 (0.64 to infinity^c^)
Tumor size, mm								
<10	34	64	‒2.32 (‒3.46 to ‒1.21)	0.53 (0.43 to 0.68)	29	20	5.26 (0.59 to 9.46)	1.45 (1.03 to 2.25)
10-19	62	146	‒4.15 (‒5.22 to ‒3.07)	0.42 (0.37 to 0.5)	60	52	2.19 (‒1.16 to 5.93)	1.15 (0.94 to 1.55)
20-49	65	208	‒9.44 (‒11.22 to ‒7.73)	0.31 (0.27 to 0.36)	82	102	‒4.71 (‒8.74 to ‒0.9)	0.8 (0.69 to 0.96)
≥50	23	80	‒18.63 (‒23.21 to ‒14.43)	0.29 (0.24 to 0.36)	21	30	‒11.11 (‒21.06 to ‒3.05)	0.7 (0.55 to 0.9)
Tumor stage								
Local	127	267	‒3.45 (‒4.18 to ‒2.75)	0.48 (0.43 to 0.53)	126	113	1.74 (‒0.9 to 4.15)	1.12 (0.95 to 1.32)
Regional	70	252	‒12.79 (‒14.64 to ‒10.99)	0.28 (0.25 to 0.31)	80	108	‒7.87 (‒12.26 to ‒3.67)	0.74 (0.65 to 0.86)
Distant	6	24	‒33.96 (‒45.74 to ‒22.66)	0.25 (0.17 to 0.36)	1	3	‒28.57 (‒65.78 to 11.67)	0.33 (0.2 to infinity^c^)
Tumor grade								
1	36	80	‒3.2 (‒4.48 to ‒2.13)	0.45 (0.37 to 0.55)	18	15	2.88 (‒3.18 to 8.81)	1.2 (0.85 to 2.03)
2	79	212	‒5.85 (‒6.91 to ‒4.6)	0.37 (0.33 to 0.43)	59	51	2.57 (‒1.42 to 6.34)	1.16 (0.93 to 1.51)
3	72	209	‒9.23 (‒10.93 to ‒7.48)	0.34 (0.3 to 0.39)	119	145	‒4.08 (‒7.09 to ‒1.2)	0.82 (0.73 to 0.94)
Estrogen receptor status								
Positive	140	393	‒6.27 (‒7.19 to ‒5.39)	0.36 (0.33 to 0.39)	90	84	1.22 (‒2.07 to 4.56)	1.07 (0.9 to 1.33)
Negative	37	87	‒5.55 (‒7.45 to ‒3.84)	0.43 (0.35 to 0.52)	97	121	‒4.68 (‒7.62 to ‒1.39)	0.8 (0.71 to 0.93)
Lymph node status								
Negative	115	247	‒3.49 (‒4.29 to ‒2.74)	0.47 (0.41 to 0.53)	124	113	1.5 (‒1.06 to 4.02)	1.1 (0.94 to 1.31)
Positive	64	236	‒13.03 (‒15.15 to ‒11.14)	0.27 (0.24 to 0.3)	71	92	‒6.48 (‒10.87 to ‒2.43)	0.77 (0.67 to 0.9)
Predict score quartile^d^								
1	25	45	‒1.92 (‒3.26 to ‒0.78)	0.56 (0.42 to 0.75)	35	22	5.94 (2.18 to 9.61)	1.59 (1.15 to 2.54)
2	28	69	‒3.94 (‒5.31 to ‒2.38)	0.41 (0.34 to 0.53)	42	42	0 (‒5.19 to 4.81)	1 (0.79 to 1.33)
3	38	104	‒6.35 (‒8.22 to ‒4.65)	0.37 (0.3 to 0.44)	36	39	‒1.38 (‒6.22 to 3.08)	0.92 (0.73 to 1.23)
4	50	165	‒11.07 (‒13.31 to ‒9.04)	0.3 (0.26 to 0.35)	47	60	‒5.96 (‒11.52 to ‒1.45)	0.78 (0.65 to 0.94)
Time between first and second breast cancer, y								
<5	97	292	‒7.64 (‒8.79 to ‒6.49)	0.33 (0.3 to 0.37)	107	134	‒5.08 (‒8.07 to ‒1.75)	0.8 (0.71 to 0.92)
5-10	85	218	‒5.72 (‒6.82 to ‒4.59)	0.39 (0.35 to 0.44)	86	82	0.81 (‒2.57 to 3.79)	1.05 (0.88 to 1.28)
≥10	24	46	‒3.03 (‒4.78 to ‒1.51)	0.52 (0.41 to 0.68)	16	13	2.97 (‒3.09 to 8.9)	1.23 (0.84 to 2.26)
Chemotherapy	85	275	‒9.77 (‒11.25 to ‒8.38)	0.31 (0.28 to 0.34)	133	164	‒4.41 (‒7.23 to ‒1.38)	0.81 (0.72 to 0.93)
Radiation therapy	106	302	‒6.3 (‒7.23 to ‒5.31)	0.35 (0.32 to 0.39)	128	145	‒2.4 (‒5.33 to 0.25)	0.88 (0.77 to 1.01)

a Absolute mortality difference=predicted ‒ observed.

b Relative mortality ratio=predicted/observed.

c The upper confidence interval was infinite due to the small number of deaths.

d This was available only for local or regional stage initial breast cancers treated by surgery and had data on tumor grade, lymph node status, and estrogen receptor status.

Sensitivity analysis by changing assumptions for the type of chemotherapy received (ie, second vs third generation) and cancer detection mode yielded similar overall results (Supplementary Tables 2 and 3, available online).

## Discussion

To our knowledge, this study is the first to evaluate model calibration and discrimination of the Predict tool among a cohort of women diagnosed with a second early-stage breast cancer. Based on a diverse cohort of women with either a first or second breast cancer, Predict performed best among those with a first estrogen receptor–positive cancer. Predict underestimated 5-year mortality in women with a second breast cancer, particularly those with estrogen receptor–positive disease. Greater underestimations in mortality were observed among non-Hispanic Black and Hispanic women; women with more aggressive tumor characteristics of the first cancer; or shorter time interval between 2 cancers. In women with estrogen receptor–negative disease, Predict overestimated mortality for a first cancer but performed well for a second cancer overall. We observed an underestimation for a second estrogen receptor–negative cancer among women who previously received chemotherapy, had a first cancer with more aggressive tumor characteristics, or had a shorter interval between first and second cancer. Predict showed good model discrimination for women with either a first or second breast cancer, irrespective of estrogen receptor status. In most clinical scenarios, the model provided a more conservative estimate by underestimating vs overestimating 5-year mortality for second cancers.

Consistent with validation studies of women diagnosed with a first breast cancer conducted mainly in White populations, Predict accurately evaluated the 5-year breast cancer mortality for a diverse population of women diagnosed with a first estrogen receptor–positive cancer but overestimated mortality for women with a first estrogen receptor–negative cancer. In a study among 45 789 cases from the Scottish Cancer Registry, Gray et al. (8) found that among women with estrogen receptor–positive diseases, the predicted 5-year mortality was only 0.5% greater than the observed one, and the AUC was 0.76. Among women diagnosed with estrogen receptor–negative breast cancer, however, a 16.6% overestimation of the 5-year mortality was observed, although the model discrimination remained excellent (AUC = 0.74). Another independent validation study using data from the Netherlands Cancer Registry reported a reliable prediction of 5-year overall survival for patients estrogen receptor–positive cancer but an underestimation of overall survival (ie, overestimation of mortality) for patients with estrogen receptor–negative cancer (7). A recent study in a New Zealand cohort also showed an overestimation of mortality among patients with estrogen receptor–negative cancer (20). Interestingly, despite possible differences in cancer screening; demographics, including race and ethnicity; and use of treatments that may affect 5-year breast cancer mortality, all these studies, including ours, reported an overestimation of mortality for estrogen receptor–negative cancers, suggesting that other unmeasured factors may also play a role.

The number of studies that have evaluated the Predict tool in a diverse population is limited. In a study among patients with early-stage breast cancer treated at the at the University of Texas MD Anderson Cancer Center, Wu et al. (21) found that the Predict tool underestimated the 5-year overall mortality for African American women but overestimated mortality for Hispanic and White women. This study, however, was based on patients from a single hospital, which limits its generalizability. Our study, which included states across the United States, showed a slight underestimation of the 5-year cancer mortality for non-Hispanic Black women with first estrogen receptor–positive cancers and greater underestimation for non-Hispanic Black and Hispanic women with second estrogen receptor–positive cancers. Predict overestimated mortality for first estrogen receptor–negative cancers across all racial and ethnic women but to a lesser degree in non-Hispanic Black women. The tool performed well for second estrogen receptor–negative cancers among non-Hispanic White women but underestimated mortality for other racial and ethnic groups. As Predict was not developed to discriminate differences in outcomes by race and ethnicity, our findings by race and ethnicity should be considered hypothesis generating.

The underestimation of breast cancer mortality we observed for both second estrogen receptor–positive and estrogen receptor–negative cancers is consistent with our prior observation that women with a second breast cancer have a higher risk of death than women with a first breast cancer, even after considering tumor characteristics of the initial and the second cancer and the time interval between 2 cancer (3,22-25). There are multiple plausible explanations for the high mortality among women with a second breast cancer. Second cancers initiated soon after therapy may be more therapy resistant (22). Our previous study also suggested that prior chemotherapy is associated with greater mortality after a second cancer (3). Persistent risk factors and comorbidities among survivors may continue to affect disease progression (26,27). Women may receive inadequate care the second time around due to various factors, such as experiencing toxicities from the first treatment, being less compliant with treatment, or facing financial stressors that limit their access to health care (28).

In this study, Predict showed reasonable model discrimination in predicting cancer mortality among women with a second cancer. These results indicate that the Predict tool could still be useful to identify groups of patients who had a higher vs lower risk of death from a second cancer, although it may not provide an accurate estimation of the absolute risk, particularly in women diagnosed with a second estrogen receptor–positive cancer.

Although we did not have detailed information about chemotherapy regimens, it is important to note that sensitivity analyses evaluating the impact of varying numbers of women who received third- vs second-generation adjuvant chemotherapy regimens did not statistically significantly alter the results. Of note, cyclin-dependent kinase inhibitors are not part of Predict.

The strengths of this study include the large number of second breast cancers; rigorous ascertainment of second cancers in the SEER registry; high-quality data on vital status and cause of death; and a diverse population that represents 28% of the US population, which allows for analysis of those from racial and ethnic minority groups. This study has several limitations, as well. First, KI-67 status and HER2 status before 2010 were not available in the SEER database. Therefore, “unknown” was entered into the model for these missing values. Second, assumptions were made for mode of detection, generation of chemotherapy, and uptake of trastuzumab. Sensitivity analyses to test different assumptions, however, yielded results consistent with the main analyses, suggesting that the results are robust. Third, chemotherapy was underreported in SEER (29). To eliminate the potential bias this issue may cause, we limited our analyses to women who received chemotherapy for estrogen receptor–negative cancers, which account for 70% of all patients with estrogen receptor–negative cancers. Another limitation is that we could not determine whether death was due to the first or second breast cancer. Therefore, it is plausible that a small number of deaths were attributed incorrectly.

In summary, this US SEER study showed that Predict largely underestimated the 5-year cancer-specific mortality in women diagnosed with a second estrogen receptor–positive breast cancer and in some subgroups of women diagnosed with a second estrogen receptor–negative breast cancer. Our findings suggest that clinicians should be cautious when applying estimates from Predict among women with a second breast cancer.

## Supplementary Material

pkad081_Supplementary_Data

## Data Availability

The data underlying this article are available from the National Cancer Institute SEER program, at https://seer.cancer.gov/.
