# Supplementary material for: Evaluation of Predict, a prognostic risk tool, after diagnosis of a second breast cancer
Source: JNCI Cancer Spectr. 2023 Sep 29;7(6):pkad081. doi: 10.1093/jncics/pkad081 (PMC10660126; doi:10.1093/jncics/pkad081)
Supplement: pkad081_Supplementary_Data [file pkad081_supplementary_data.pdf]

Supplementary Table 1. Area under the curve (AUC) of PREDICT model by race and ethnicity for first and second breast cancer by estrogen receptor (ER) status.

	First breast cancer		Second breast cancer	
	ER-positive	ER-negative	ER-positive	ER-negative
Non-Hispanic White	0.817	0.754	0.727	0.723
Non-Hispanic Black	0.806	0.751	0.796	0.724
Hispanic	0.813	0.753	0.802	0.775
Other[1]	0.831	0.744	0.697	0.755

[1]Other included Non-Hispanic Asian and Pacific Islander, Non-Hispanic American Indian and Alaska Native

Supplementary Table 2. Model performance when 50% of breast cancers were detected by screening.

	First breast cancer						Second breast cancer				
	Total number of cases (N)	Predicted number of deaths (P)	Observed number of deaths (O)	Absolute mortality difference (95% CI) [1]	Relative mortality ratio (95%CI) [2]		Total number of cases (N)	Predicted number of deaths (P)	Observed number of deaths (O)	Absolute mortality difference (95% CI) [1]	Relative mortality ratio (95%CI) [2]
ER-positive	303837	13734	13855	-0.04 (-0.11,0.03)	0.99 (0.98,1.01)		5606	215	556	-6.08 (-6.81,-5.34)	0.39 (0.36,0.42)
ER-negative	53367	11768	9343	4.54 (4.27,4.86)	1.26 (1.24,1.28)		1123	210	229	-1.69 (-3.99,0.16)	0.92 (0.83,1.01)

ER: estrogen receptor
[1]Absolute mortality difference=P-O
[2]Relative mortality ratio=P/O

Supplementary Table 3. Model performance when chemotherapy was 2nd or 3rd generation for all calendar years.

	First breast cancer						Second breast cancer				
	Total number of cases (N)	Predicted number of deaths (P)	Observed number of deaths (O)	Absolute mortality difference (95% CI) [1]	Relative mortality ratio (95%CI) [2]		Total number of cases (N)	Predicted number of deaths (P)	Observed number of deaths (O)	Absolute mortality difference (95% CI) [1]	Relative mortality ratio (95%CI) [2]
Chemotherapy was 2nd generation for all calendar years											
ER-positive	303837	14123	13855	0.09 (0.02,0.16)	1.02 (1,1.04)		5606	219	556	-6.01 (-6.73,-5.25)	0.39 (0.37,0.43)
ER-negative	53367	13000	9343	6.85 (6.58,7.17)	1.39 (1.37,1.42)		1123	241	229	1.07 (-1.18,2.97)	1.05 (0.95,1.16)
Chemotherapy was 3rd generation for all calendar years											
ER-positive	303837	12776	13855	-0.36 (-0.43,-0.28)	0.92 (0.91,0.94)		5606	205	556	-6.26 (-6.98,-5.51)	0.37 (0.34,0.4)
ER-negative	53367	11159	9343	3.4 (3.13,3.71)	1.19 (1.18,1.22)		1123	207	229	-1.96 (-4.27,-0.07)	0.9 (0.81,1)

ER: estrogen receptor
[1]Absolute mortality difference=P-O
[2]Relative mortality ratio=P/O
